# Associations between Safety from Crime, Cycling, and Obesity in a Dutch Elderly Population: Results from the Longitudinal Aging Study Amsterdam

**DOI:** 10.1155/2012/127857

**Published:** 2012-02-22

**Authors:** Stef P. J. Kremers, Gert-Jan de Bruijn, Tommy L. S. Visscher, Dorly J. H. Deeg, G. C. Fleur Thomése, Marjolein Visser, Willem van Mechelen, Johannes Brug

**Affiliations:** ^1^Department of Health Promotion, School for Nutrition, Toxicology and Metabolism (NUTRIM), Maastricht University, P.O. Box 616, 6200 MD Maastricht, The Netherlands; ^2^Amsterdam School of Communication Research, 1012 CX Amsterdam, The Netherlands; ^3^Research Centre for the Prevention of Overweight, VU-Windesheim, 8000 GB Zwolle, The Netherlands; ^4^EMGO Institute, VU University Medical Center, 1081 BT Amsterdam, The Netherlands; ^5^Department of Sociology, Faculty of Social Sciences, VU University, 1081 HV Amsterdam, The Netherlands; ^6^Department of Public and Occupational Health and EMGO Institute, VU University Medical Center, 1081 BT Amsterdam, The Netherlands

## Abstract

The objective of this cross-sectional study was to investigate differences in associations between crime rates, cycling, and weight status between people living in low and high socioeconomic status (SES) neighbourhoods. In total, 470 participants in the Longitudinal Aging Study Amsterdam were included (age: 63–70 y). Body height and weight were measured using a stadiometer and calibrated weight scale, respectively. Cycling behaviour was assessed in a face-to-face interview, and neighbourhood crime rates were assessed using data from police reports. Men residing in high SES neighbourhoods cycled more than males residing in low SES neighbourhoods. Cycling was negatively related to crime rates among both men and women living in low SES neighbourhoods. Among men living in low SES neighbourhoods, more cycling was associated with lower BMI. Interventions aiming to prevent obesity in older people may consider aiming at increasing bicycle use in lower SES neighbourhoods, but neighbourhood safety issues should be considered.

## 1. Introduction

Obesity prevalence rates have increased worldwide in recent decades [1–4] and represent a major public health problem [5–8]. Absolute risk for disease associated with obesity is highest in the elderly [9]. Although genetic factors are thought to influence the individual susceptibility to obesity [10], environmental and behavioural factors are arguably of greater importance to the current obesity epidemic [11]. Weight gain occurs when energy intake exceeds energy expenditure over a period of time, leading to a positive energy balance. With respect to energy expenditure, cycling is more energy intensive per unit of time than walking [12], and it is a common mode of active transport in some countries with favorable city planning and infrastructure, such as The Netherlands and the Scandinavian countries [13]. In The Netherlands, cycling is also relatively common among elderly. Approximately 55% of the Dutch 55–75-year-old population cycles more than one hour per week on average [14]. Cycling is deemed important in the prevention of obesity [12]. About 75% of Dutch 65–74 year-olds is overweight or obese [15]. 

Evidence exists that individual socioeconomic status (SES), based on such indicators as educational level and income level, is associated with obesity [16–19]. Furthermore, neighbourhood SES has been found to be associated with overweight prevalence rates [20, 21]. A large body of evidence links specific environmental factors within these neighbourhoods with levels of physical activity [21–28]. One such environmental factor is neighbourhood safety, but research on the association between safety from crime and physical activity among older adults revealed mixed results [29]. Some studies supported the relationship between crime-related safety and reduced physical activity of older adults [30–33], but others found no association [34–36]. Nonetheless, Foster and Giles-Corti [29] concluded that the current evidence suggests that, particularly for women and older adults, crime-related safety may constrain physical activity. Studies that specifically examine the relationship between objectively assessed safety from crime and cycling among older adults are lacking, however.

The aim of the present study was twofold. First, we investigated differences in obesity prevalence rates and crime rates between low and high SES neighbourhoods. Second, we explored the associations between crime rates with cycling and BMI in a sample of Dutch elderly.

## 2. Subjects and Methods

### 2.1. Study Sample

Data for the present study were available from the fourth data collection of the Longitudinal Aging Study Amsterdam (LASA) in 2001/2002. LASA is an ongoing multidisciplinary longitudinal study of predictors and consequences of changes in autonomy and well-being in the aging population. The design of LASA has been described in full detail elsewhere [37–39]. Briefly, a nationally representative random sample of older adults stratified by age and sex was drawn from population registers of 11 municipalities in three culturally distinct geographical areas in The Netherlands. Subjects who completed the fourth data collection cycle in 2001/2002 were included in the present study (*N* = 1691). As the association between BMI and adiposity changes with aging [40, 41], subjects older than 70 years of age were excluded from the present analyses. The study was approved by the Ethics Review Board of the VU University Medical Center, and informed consent was obtained from all respondents.

### 2.2. Neighbourhood and Individual Socioeconomic Status

Neighbourhood SES was assessed using data from Statistics Netherlands on (1) average income per household, (2) percentage of households with low income, (3) percentage of unemployed people, and (4) percentage of households with low educational level. Factor loadings for these variables were all >.70. From these factors, a single score was computed into a social status neighbourhood score (SSNS), in which a higher score corresponded with a higher SES of the neighbourhood. These data were linked to participants using their four-digit postal code. A median split was used to categorize respondents in either low (*n* = 235) or high (*n* = 235) SES neighbourhoods.

Educational level and personal income were used as individual indicators of SES using closed questions. Respondents were asked to report on their highest attained education, ranging from (1) elementary school not completed to (9) university education. Monthly income in guilders was asked using categories ranging from approximately (1) (€450–€550) to (12) >€2.250, and then quartiles were used to categorize respondents. For educational level, responses were recoded into (1) no education (*n* = 34), (2) elementary and lower education (*n* = 207), (3) secondary and intermediate education (*n* = 147), and (4) higher education (*n* = 82).

### 2.3. Crime Rates

Data on crime rates were available from “misdaadmeter” [42], in which the frequency of six crimes (raid, theft/burglary/housebreaking, theft from car, murder and threat/robbery) is assessed annually at the municipal level using data from police reports. For each municipality, the frequency of these crime rates was divided by population of that municipality, and this resulting score was divided by 1000. These scores were then standardised and summed and ranged from 0 to 100, with a higher score indicating a higher crime rate. Respondents' postal code was used to match crime rates with the participants, based on the municipal code by Statistics Netherlands [43].

### 2.4. Obesity

Body height was measured to the nearest 0.001 m using a stadiometer, while body weight was measured to the nearest 0.1 kg using a calibrated scale. When respondents wore a corset or clothes during the measurement, 1 or 2 kg, respectively, were subtracted from the measured body weight. BMI was computed as body weight (kg)/body height (m)^2^, with BMI ≥ 30 kg/m^2^ indicating obesity.

### 2.5. Cycling

In a face-to-face structured interview based on a validated questionnaire, named LAPAQ (LASA Physical Activity Questionnaire) [44], respondents were asked to report the frequency and duration of physical activities during the two weeks preceding the interview. The answers to the three questions regarding cycling were analyzed for the purpose of the present study. These questions were: Did you ride your bike in the past two weeks? On how many days of the past two weeks did you ride a bike? and How long, on average, did you ride a bike on these occasions? Multiplying frequency and duration and dividing that score by 14 computed an average time in minutes per day.

### 2.6. Statistical Analyses

Data was analysed with SPSS 18.0. First, logistic regression analysis was used to examine the relationship between gender and social status neighbourhood score (SSNS) with obesity. Both unadjusted (crude) and adjusted (for age, personal educational level, and personal income level) odds ratios were computed. Since neighbourhood deprivation has been reported to be more strongly related with overweight in females than in males [16, 21], gender-stratified analyses were also conducted. Finally, within low and high SES neighbourhoods, gender-stratified linear regression analyses were conducted to explore associations between crime rates with cycling and BMI, while adjusting for age, personal educational level, and personal income level.

## 3. Results

Deletion of cases with no data on postal code and/or neighbourhood SES (*n* = 2) and income (*n* = 25) left a final sample of 470 participants (230 men and 240 women). Mean age was 66.5 years (SD = 2.3: range 63–70 years), and mean BMI was 27.5 (SD = 4.1) (Table 1). Men had significantly lower BMI (26.8 versus 28.1) than women. Non-significant differences in BMI were found between low and high SES neighbourhood residents. Men cycled significantly more (15.4 (range 0–107.1) versus 11.0 (range 0–102.9) minutes per day) than women. In total, 88.6% of the population engaged in any cycling (88.5% males; 88.7% females). In low SES neighbourhoods, significantly more crimes (29.8 versus 14.7) were reported compared to high SES neighbourhoods. Men residing in these high SES neighbourhoods cycled significantly more (18.5 versus 12.4 minutes per day) than men residing in low SES neighbourhoods.

Table 2 shows that women were significantly more likely to be obese than men (OR = 2.58, 95% CI = 1.60–4.16). Neighbourhood SES was not significantly associated with obesity (OR =  .76, 95% CI =  .48–1.18), but gender-stratified analyses revealed that men living in a high SES neighbourhood were less likely to be obese (OR =  .38, 95% CI =  .17–.85) than men living in a low SES neighbourhood. A nonsignificant association was found between neighbourhood SES and obesity among women (OR = 1.09, 95% CI =  .62–1.90).

Table 3 shows the results of the regression analyses. More cycling was found to be associated with lower BMI among men (ß = −.290, *P* = .002) and women (ß = −.184, *P* = .050) living in low SES neighbourhoods. Among men residing in low SES neighbourhoods, higher crime rates were significantly associated with higher BMI (ß =  .333, *P* < .001). Higher crime rates were significantly associated with less cycling in both men (ß = −.242, *P* = .011) and women (ß = −.193, *P* = .041) residing in low SES neighbourhoods.

## 4. Discussion

The purpose of the present study was to investigate associations between crime rates, cycling, and weight status among Dutch elderly living in low and high SES neighbourhoods. In line with an earlier study in The Netherlands [45], our results showed that more cycling was associated with lower BMI, especially among residents of low SES neighbourhoods. Replicating earlier results [46, 47], our findings further showed that residents of low SES neighbourhoods cycled less than residents of high SES neighbourhoods, especially among men. In line with earlier findings [48], men cycled more than women. One possible explanation for this gender difference in our study may be that lifestyle changes in the elderly have a stronger effect on men than on women, for instance because of retirement of formerly active jobs [45, 48].

Higher crime rates were associated with less cycling, especially among residents of low SES neighbourhoods. This result underlines the conclusion of the review by Foster and Giles-Corti [29] regarding the potential physical activity restraining influence of neighbourhood crime among older populations, and it adds the specific transportation mode of cycling to the evidence base. An interesting pattern emerged from our analyses. Men living in low SES neighbourhoods had an almost threefold increased chance of being obese when compared with men living in high SES neighbourhoods. Furthermore, they cycled on average about 45 minutes per week less than men living in high SES neighbourhoods. In these men, less cycling was significantly associated with higher crime rates and, importantly, significantly higher crime rates were observed in low SES neighbourhoods. The cross-sectional nature of the data for the present study withheld us from conducting mediation analyses regarding the potential mediating role of cycling in the crime-BMI relationship, however. Future longitudinal or experimental studies could provide further insights in this respect.

The results of our study should be viewed in light of several limitations. First, height and weight were used to determine BMI and obesity. Due to a decline in stature, use of height commonly overestimates BMI in the elderly and obesity in this age group is probably better reflected by waist circumference than by BMI [36, 37, 49]. Second, cycling behaviour was assessed based on self-reports. Although interviews are regarded as more valid than questionnaires [50], self-reported behaviour is known to be sensitive to over- and underreporting [51]. Our measure did not include the type of cycling behaviour (e.g., cycling for transport versus cycling for leisure), however, making it impossible to relate type of cycling behaviour to our explanatory variables. Third, we isolated crime rates from other potentially important (environmental) determinants of cycling. Several studies have indicated that environmental factors such as availability of green areas and recreational spaces [52], aesthetics and proximity to facilities [53] are associated with physical activity in The Netherlands, as well as elsewhere [22, 54–56]. In addition, Ball and colleagues [34] showed that neighbourhood-level social environmental variables may be of greater importance than safety from crime for engaging in physical activity. Fourth, selection bias may have occurred since nonresponse has been selective for the older and less healthy subjects. Paradoxically, oversampling in the most vulnerable strata of the older population (i.e., specifically targeting the inclusion of sufficient older males and those in more urbanized areas) may have led to a higher nonresponse. Finally, we applied secondary analyses on cross-sectional data, implying that our results are somewhat outdated and no causal patterns between the variables studies could be identified. A major strength of our study was the use of objective measures for height and weight and observed crime rates. Reported body weight and height would probably have led to attenuated relations since body weight is especially underreported in obese persons.

To conclude, the present study provided indications of the negative association between cycling and crime rates in low SES neighbourhoods. Interventions aiming to increase physical activity or to prevent obesity in older people may consider aiming at increasing bicycle use in lower SES neighbourhoods, but such interventions should consider neighbourhood safety issues.

## Figures and Tables

**Table 1:** Odds ratios and 95% confidence interval for obesity, based on gender and social status neighbourhood score (SSNS).

	*N*	Age (SD)	*N* = obese (%)	Crude OR	95% CI	Adjusted OR*	95% CI
Total	470	66.5 (2.3)	108 (23.0)				

Gender							
Men	230	66.4 (2.4)	32 (13.9)	1		1	
Women	240	66.6 (2.2)	76 (31.7)	2.00	1.24–3.22	2.58	1.60–4.16
*P* value		.53			<.01	<.001	

SNSS							
Low status	235	66.6 (2.2)	60 (26.1)	1		1	
High status	235	66.4 (2.4)	48 (20.0)	.94	.71–1.25	.76	.48–1.18
*P* value		.23		.67		.22	

Men							
Low SSNS	115	66.5 (2.3)	22 (19.1)	1		1	
High SSNS	115	66.3 (2.4)	10 (8.7)	.72	.47–1.09	.38	.17–.85
*P* value		.48		.12		.02	

Women							
Low SSNS	120	66.7 (2.0)	38 (31.7)	1		1	
High SSNS	120	66.4 (2.3)	38 (31.7)	1.18	.79–1.78	1.09	.62–1.90
*P* value		.33		.42		.78	

*odds ratio, adjusted for age, personal educational level, and personal income level.

**Table 2:** Mean scores and standard deviations for BMI, cycling (minutes per day), social status neighbourhood score (SSNS), and crime rates.

	*N*	BMI (SD)	Cycling (SD)	SSNS (SD)	Crime (SD)
Total	470	27.5 (4.1)	13.2 (23.7)	−.09 (.88)	22.1 (27.0)

Gender					
Men	230	26.8 (3.3)	15.4 (28.5)	−.11 (.92)	21.9 (26.4)
Women	240	28.1 (4.8)	11.0 (17.7)	−.06 (.85)	22.3 (27.7)
*P* value		.001	.042	.492	.872

SNSS					
Low status	235	27.6 (4.3)	10.9 (20.5)	−.71 (.80)	29.8 (30.8)
High status	235	27.4 (4.0)	15.3 (26.2)	.51 (.43)	14.7 (20.3)
*P* value		.608	.041	<.001	<.001

Obesity					
Nonobese	362	25.7 (2.5)	14.2 (25.4)	−.05 (.85)	21.0 (26.2)
Obese	108	33.4 (2.9)	9.5 (16.3)	−.20 (1.00)	25.7 (29.5)
*P* value		<.001	.066	.134	.116

Men					
Low SSNS	115	27.1 (3.6)	12.4 (23.9)	−.76 (.83)	28.4 (30.6)
High SSNS	115	26.5 (2.9)	18.5 (32.3)	.53 (.41)	14.9 (19.2)
*P* value		.162	.007	<.001	<.001

Women					
Low SSNS	120	27.9 (4.9)	10.3 (19.0)	−.63 (.77)	29.6 (31.1)
High SSNS	120	28.3 (4.7)	11.7 (16.4)	.51 (.45)	15.0 (21.6)
*P* value		.532	.268	<.001	<.001

**Table 3:** Unstandardized regression coefficients, standard errors, and standardized regression coefficients from the regression analyses, in men and women residing in low and high socio-economic status neighbourhoods.

	Men low status			Men high status			Women low status			Women high status		
	Unstand.	SE	Stand.	Unstand.	SE	Stand.	Unstand.	SE	Stand.	Unstand.	SE	Stand.
Cycling-BMI	−.052	.016	−.290**	−.017	.021	−.076	−.023	.011	−.184*	−.015	.012	−.117
Crime-BMI	.039	.011	.333***	.014	.014	.094	.012	.015	.077	−.029	.021	−.138
Crime-cycling	−.005	.002	−.242*	−.005	.003	−.143	−.004	.002	−.193*	−.004	.003	−.136

*Note*. All analyses were adjusted for age, personal educational level, and personal income level. Unstand.: unstandardized regression coefficient; SE: standard error; Stand.: standardized regression coefficient.

*P < .05; **P < .01; ***P < .001.

## References

[1] Flegal KM, Carroll MD, Ogden CL, Johnson CL (2002). Prevalence and trends in obesity among US adults, 1999-2000. *Journal of the American Medical Association*.

[2] Ogden CL, Flegal KM, Carroll MD, Johnson CL (2002). Prevalence and trends in overweight among US children and adolescents, 1999-2000. *Journal of the American Medical Association*.

[3] Seidell JC, Verschuren WMM, Kromhout D (1995). Prevalence and trends of obesity in The Netherlands 1987–1991. *International Journal of Obesity*.

[4] Sundquist K, Qvist J, Johansson SE, Sundquist J (2004). Increasing trends of obesity in Sweden between 1996/97 and 2000/01. *International Journal of Obesity*.

[5] Flegal KM (2000). Obesity, overweight, hypertension, and high blood cholesterol: the importance of age. *Obesity Research*.

[6] Garrison RJ, Higgins MW, Kannel WB (1996). Obesity and coronary heart disease. *Current Opinion in Lipidology*.

[7] Peeters A, Barendregt JJ, Willekens F (2003). Obesity in adulthood and its consequences for life expectancy: a life-table analysis. *Annals of Internal Medicine*.

[8] Visscher TLS, Rissanen A, Seidell JC (2004). Obesity and unhealthy life-years in adult finns: an empirical approach. *Archives of Internal Medicine*.

[9] Seidell JC, Nooyens AJ, Visscher TLS (2005). Cost-effective measures to prevent obesity: epidemiological basis and appropriate target groups. *Proceedings of the Nutrition Society*.

[10] Kumanyika S, Jeffery RW, Morabia A, Ritenbaugh C, Antipatis VJ (2002). Obesity prevention: the case for action. *International Journal of Obesity*.

[11] Hill JO, Wyatt HR, Melanson EL (2000). Genetic and environmental contributions to obesity. *Medical Clinics of North America*.

[12] Shephard RJ (2008). Is active commuting the answer to population health?. *Sports Medicine*.

[13] Bere E, Oenema A, Prins RG, Seiler S, Brug J (2011). Longitudinal associations between cycling to school and weight status. *International Journal of Pediatric Obesity*.

[14] Continuous research lifesituation. http://www.scp.nl.

[15] van Bakel AM, Zantinge EM (2010). How many people are overweight or underweight?. *Volksgezondheid Toekomst Verkenning*.

[16] Sobal J, Stunkard AJ (1989). Socioeconomic status and obesity: a review of the literature. *Psychological Bulletin*.

[17] Ball K, Crawford D (2005). Socioeconomic status and weight change in adults: a review. *Social Science and Medicine*.

[18] Ball K, Mishra GD, Crawford D (2003). Social factors and obesity: an investigation of the role of health behaviours. *International Journal of Obesity*.

[19] Wamala SP, Wolk A, Orth-Gomér K (1997). Determinants of obesity in relation to socioeconomic status among middle-aged Swedish women. *Preventive Medicine*.

[20] Ellaway A, Anderson A, Macintyre S (1997). Does area of residence affect body size and shape?. *International Journal of Obesity*.

[21] van Lenthe FJ, Mackenbach JP (2002). Neighbourhood deprivation and overweight: the GLOBE study. *International Journal of Obesity*.

[22] Booth SL, Sallis JF, Ritenbaugh C (2001). Environmental and societal factors affect food choice and physical activity: rationale, influences, and leverage points. *Nutrition Reviews*.

[23] Brug J, van Lenthe FJ, Kremers SPJ (2006). Revisiting kurt lewin: how to gain insight into environmental correlates of obesogenic behaviors. *American Journal of Preventive Medicine*.

[24] Humpel N, Owen N, Iverson D, Leslie E, Bauman A (2004). Perceived environment attributes, residential location, and walking for particular purposes. *American Journal of Preventive Medicine*.

[25] Li F, Fisher KJ, Bauman A (2005). Neighborhood influences on physical activity in middle-aged and older adults: a multilevel perspective. *Journal of Aging and Physical Activity*.

[26] Wendel-Vos W, Droomers M, Kremers S, Brug J, Lenthe FVan (2007). Potential environmental determinants of physical activity in adults: A systematic review. *Obesity Reviews*.

[27] Diez-Roux AV, Javier Nieto F, Muntaner C (1997). Neighborhood environments and coronary heart disease: a multilevel analysis. *American Journal of Epidemiology*.

[28] Turrell G, Haynes M, Burton NW (2010). Neighborhood disadvantage and physical activity: baseline results from the HABITAT multilevel longitudinal study. *Annals of Epidemiology*.

[29] Foster S, Giles-Corti B (2008). The built environment, neighborhood crime and constrained physical activity: an exploration of inconsistent findings. *Preventive Medicine*.

[30] Li F, Fisher KJ, Brownson RC, Bosworth M (2005). Multilevel modelling of built environment characteristics related to neighbourhood walking activity in older adults. *Journal of Epidemiology and Community Health*.

[31] Mota J, Lacerda A, Santos MP, Ribeiro JC, Carvalho J (2007). Perceived neighborhood environments and physical activity in an elderly sample. *Perceptual and Motor Skills*.

[32] Piro FN, Nœss Ø, Claussen B (2006). Physical activity among elderly people in a city population: the influence of neighbourhood level violence and self perceived safety. *Journal of Epidemiology and Community Health*.

[33] Wilcox S, Bopp M, Oberrecht L, Kammermann SK, McElmurray CT (2003). Psychosocial and perceived environmental correlates of physical activity in rural and older African American and white women. *Journals of Gerontology B*.

[34] Ball K, Cleland VJ, Timperio AF, Salmon J, Giles-Corti B, Crawford DA (2010). Love thy neighbour? Associations of social capital and crime with physical activity amongst women. *Social Science and Medicine*.

[35] Booth ML, Owen N, Bauman A, Clavisi O, Leslie E (2000). Social-cognitive and perceived environment influences associated with physical activity in older Australians. *Preventive Medicine*.

[36] Lim K, Taylor L (2005). Factors associated with physical activity among older people—a population-based study. *Preventive Medicine*.

[37] Deeg DJH, Knipscheer CPM, van Tilburg W (1993). *Autonomy and Well-Being in the Aging Population: Concepts and Design of the Longitudinal Aging Study Amsterdam*.

[38] Huisman M, Poppelaars J, van der Horst M (2011). Cohort profile: the longitudinal aging study Amsterdam. *International Journal of Epidemiology*.

[39] Beekman ATF, Deeg DJH, van Tilburg T, Smit JH, Hooijer C, van Tilburg W (1995). Major and minor depression in later life: a study of prevalence and risk factors. *Journal of Affective Disorders*.

[40] Gallagher D, Visser M, Sepúlveda D, Pierson RN, Harris T, Heymsfieid SB (1996). How useful is body mass index for comparison of body fatness across age, sex, and ethnic groups?. *American Journal of Epidemiology*.

[41] Seidell JC, Visscher TLS (2000). Body weight and weight change and their health implications for the elderly. *European Journal of Clinical Nutrition*.

[42] http://www.ad.nl/misdaadmeter.

[43] Statistics Netherlands (2003). *Quarter and Neighbourhood Register 2001*.

[44] Stel VS, Smit JH, Pluijm SMF, Visser M, Deeg DJH, Lips P (2004). Comparison of the LASA physical activity questionnaire with a 7-day diary and pedometer. *Journal of Clinical Epidemiology*.

[45] Nooyens ACJ, Visscher TLS, Jantine Schuit A (2005). Effects of retirement on lifestyle in relation to changes in weight and waist circumference in Dutch men: a prospective study. *Public Health Nutrition*.

[46] Parks SE, Housemann RA, Brownson RC (2003). Differential correlates of physical activity in urban and rural adults of various socioeconomic backgrounds in the United States. *Journal of Epidemiology and Community Health*.

[47] Yen IH, Kaplan GA (1998). Poverty area residence and changes in physical activity level: evidence from the Alameda County Study. *American Journal of Public Health*.

[48] Krizek KJ, Johnson PJ, Tilahun N (2005). *Gender Differences in Bicycling Behaviour and Facility Preferences. Research on Women's Issues, Report of a Conference*.

[49] Visscher TLS, Seidell JC, Molarius A, van der Kuip D, Hofman A, Witteman JCM (2001). A comparison of body mass index, waist-hip ratio and waist circumference as predictors of all-cause mortality among the elderly: the Rotterdam study. *International Journal of Obesity*.

[50] Dishman RK, Washburn RA, Schoeller DA (2001). Measurement of physical activity. *Quest*.

[51] Schwarz N (1999). Self-reports: how the questions shape the answers. *American Psychologist*.

[52] Wendel-Vos GCW, Schuit AJ, de Niet R, Boshuizen HC, Saris WHM, Kromhout D (2004). Factors of the physical environment associated with walking and bicycling. *Medicine and Science in Sports and Exercise*.

[53] van Lenthe FJ, Brug J, MacKenbach JP (2005). Neighbourhood inequalities in physical inactivity: the role of neighbourhood attractiveness, proximity to local facilities and safety in the Netherlands. *Social Science and Medicine*.

[54] Owen N, Humpel N, Leslie E, Bauman A, Sallis JF (2004). Understanding environmental influences on walking: review and research agenda. *American Journal of Preventive Medicine*.

[55] de Bourdeaudhuij I, Sallis JF, Saelens BE (2003). Environmental correlates of physical activity in a sample of Belgian adults. *American Journal of Health Promotion*.

[56] Sugiyama T, Francis J, Middleton NJ, Owen N, Giles-CortI B (2010). Associations between recreational walking and attractiveness, size, and proximity of neighborhood open spaces. *American Journal of Public Health*.

